# The Nutritional Quality of Food Donated to a Western Australian Food Bank

**DOI:** 10.3390/nu16040509

**Published:** 2024-02-11

**Authors:** Sharonna Mossenson, Roslyn Giglia, Claire E. Pulker, Satvinder S. Dhaliwal, Miranda Chester, Ruby Bigwood, Christina M. Pollard

**Affiliations:** 1 School of Population Health, Curtin University, Kent St, Perth 6102, Australia; 2 Foodbank of Western Australia, Perth Airport, Perth 6105, Australia; 3 East Metropolitan Health Service, Murray Street, Perth 6004, Australia; 4 Duke-NUS Medical School, National University of Singapore, Singapore 169857, Singapore; 5 Institute for Research in Molecular Medicine (INFORMM), Universiti Sains Malaysia, Minden 11800, Pulau Pinang, Malaysia; 6 Office of the Provost, Singapore University of Social Sciences, Singapore 599494, Singapore; 7 Enable Institute, Curtin University, Kent St, Perth 6102, Australia; 8 Curtin Health Innovation Research Institute (CHIRI), Curtin University, Kent St, Perth 6102, Australia

**Keywords:** food banks, donation/s, food insecurity, nutritional quality, nutrition classification schemes, dietary guidelines, NOVA, nutrition, ultra-processed food

## Abstract

Food banks provide an indispensable service to people experiencing severe food insecurity. Food banks source donations from across the food system; however, the food redistributed to clients across the developed world is nutritionally poor. This, together with the increasing prevalence of diet-related diseases and food insecurity, has prompted a focus on nutritional quality. Despite more food being distributed via food banks in Australia, the nutritional quality of donated food remains unreported. This study analyzed all food (84,996 kg (1216 products)) donated to Foodbank WA over a 5-day period using diet-, food-, and nutrient-based nutrition classification schemes (NCSs). A total of 42% (27% of total weight) of donated food products were deemed ‘unsuitable’ and 19% (23% by weight) were ‘suitable’ according to all NCSs. There was no agreement on 39% of products (50% by weight). Overall, NOVA and the Healthy Eating Research Nutrition Guidelines (HERNG) (κ = 0.521) had the highest level of agreement and the ADGs and HERNGs the lowest (κ = 0.329). The findings confirm the poor nutritional quality of food donated to food banks and the need to work with donors to improve the food they donate. Fit-for-purpose nutrition guidelines are urgently needed for Australian food banks to support them in providing nutritious food to their vulnerable clients.

## 1. Introduction

Food banks have been one of the fastest growing charitable industries in the developed world since the 1980s [1]. Although long regarded as emblematic of government inaction in addressing the underlying causes of poverty, food banks provide an indispensable service to people experiencing severe and often chronic food insecurity [2,3]. The food system spans production, aggregation, processing, distribution, consumption, and disposal of food [4], and food banks source donations from across this system, including from retailers, manufacturers, distributors, and growers [5,6,7]. Food banks’ food supply is often supplemented with funding from government (e.g., United States (US) federal commodity and food assistance programs), nongovernment, and philanthropic sources [5,6,7]. The majority of the food supply [5,8,9] is via surplus food redistribution (SFR), that is, donations from the retail food sector that are surplus to requirements, close to expiry, or rejected for sale due to mislabeling, end of line, an imperfect size/shape, or food that does not appeal to consumer tastes and preferences [10].

Food banks are the ‘back-line’ organizations who recover, transport, warehouse, and redistribute donated food to ‘front-line’ agencies who provide food directly to clients (e.g., food pantries or food shelves in the US). In Canada, Australia, the United Kingdom (UK), and some parts of Europe (Germany, the Netherlands), food banks perform both front- and back-line functions [7,11,12], and this is the type of food bank that is the focus of this study. This complex network, which also includes congregate meal sites and food rescue organizations, is collectively referred to as the ‘charitable food system’ (CFS) [13].

Aiming to distribute food to as many people as possible as quickly as possible [14], food banks measure their success based on the weight of food they distribute [9,15]. For example, in 2021, the Foodbank of Western Australia sourced 43 million kilograms of food, representing 7.8 million meals [16]. The weight-based performance indicator emphasizes food quantity over quality, making it difficult for both donors and food banks to prioritize healthier food as ‘*retailers are incentivized to give more, not better*’ [17]. Weight is at odds with policies to eliminate unhealthy, heavy-weight products such as sugar-sweetened beverages (or soda in the US) [18].

Research suggests that the food distributed through food banks across the developed world continues to be nutritionally poor [19,20]. The provision of low-quality food is likely a reflection of the broader food system and donors’ priorities [21,22] and also results in an unpredictable and variable food supply [23]. This is further complicated by increasing demand for food and the finite capacity of food banks [24], which are some of the many operational challenges food banks confront daily. This is concerning given some clients use food banks as frequently as grocery stores [25] and the food sourced from food banks can comprise over half of a client’s total daily dietary intake [26,27]. The major predictor of food bank patronage is financial hardship due to low, limited, or insecure income [28,29,30]. People who are living alone, single parents, the un- or underemployed, social welfare benefit recipients, and people with a disability or mental illness are more likely to experience food insecurity and require food relief [31,32,33]. Poor diet quality and obesity are associated with using food banks [34,35], which compounds existing nutritional vulnerabilities and disproportionately high rates of diet-related chronic disease and poor mental health among clients [35,36].

The increasing prevalence of obesity, diet-related disease, and food insecurity has focused attention on the quality of food distributed by food banks [37], and the sector has a collective interest in moving toward the provision of healthier food [38]. Although typically falling outside the remit of research which examines the impact of local food environments on eating behavior [39], there is increasing recognition of the influential role food banks play in shaping the diets of their clients [38]. There are many policy, system, and environment (PSE) interventions, including nutrition policies, choice architecture, and nutrition education [22], that aim to improve and support the health status of clients accessing the CFS. Food banks’ support for PSE initiatives continues to grow as clients prioritize healthier food options [25,33,40,41].

Initiatives and approaches to tracking and improving the quality of food banks’ inventories have increased over the last few decades [13,22]. Over half of US food banks track the nutritional quality of their food inventories utilizing various nutrition classification systems (NCSs) based on diet, food, or nutrient profiling [8]. Collectively, these approaches evaluate the nutritional profile of inventory to help guide procurement decisions [8], inform policy, improve the quality of supplies [42], and, in turn, positively influence client selections [43].

Diet-based approaches incorporate concepts of overall diet quality, variety, and adequacy and include indices derived from national food-based dietary guidelines [44]. Hoisington (2011) defined the nutritional quality of foods distributed by US food banks based on the availability of food per food group [45]. Other examples [46,47,48,49,50] translate the holistic diet-based approach to reductionist nutrient profiling, which focuses on the effects of specific beneficial or harmful nutrients and food components on metabolic processes or health outcomes [44]. Using an algorithm or index, the nutritional value of food distributed (by weight) is scored against nutrient density and alignment with national dietary guidelines. The Healthy Eating Research Nutrition Guidelines (HERNGs) [51] use nutrient profiling (for saturated fat, salt, and sugars) to assess inventory quality and rank foods across 11 food categories into three tiers (‘choose often’, ‘choose sometimes’, and ‘choose rarely’). The HERNGs are consensus guidelines used across the CFS in the US [13,51]. Food-based classifications are informed by evidence of a food’s structure or composition and diet-related disease risk [44]. For example, the NOVA food classification system which categorizes food according to the extent and purpose of the processing it undergoes [52] has been used in conjunction with nutrient criteria to assess the nutritional quality of two food pantries in the US [53]. Nutrition classification systems should be context-specific and consistent with national dietary guidelines; therefore, methods established for the CFS in other countries may not translate appropriately in Australia [44].

To date, most food bank inventory classification has occurred in the US, likely owing to its long history of food banking, organizational structures, and government funding [5,6,14]. Despite having established nationwide operations for over 40 years [7], and a commitment to nutrition education and food literacy initiatives [54], there are no reports of Australian food banks adopting a systematic process to assessing the nutritional quality of food donations. Consequently, a comprehensive overview of the nutritional quality of foods donated to Australian food banks is absent from the literature. This gap in intelligence means food banks do not have the capacity to identify, assess, or improve food inventory quality, nor is there any incentive for donors to be transparent and accountable about the types of food they donate. Following the development of an audit protocol [55], this paper describes the assessment of the nutritional quality of donated food at an Australian food bank, the Foodbank of Western Australia (FBWA), using existing diet-, food-, and nutrient-based classification measures. This case study utilizes data previously collected from FBWA, which is part of the largest food banking organization in Australia. The application of three NCSs and the determination of their level of agreement contribute to the understanding of the feasibility and suitability of each scheme for monitoring and assessing the nutritional quality of donated food.

## 2. Materials and Methods

Data collected during a once-off five-day audit of food donated to FBWA were used to assess the nutritional quality of donated food. The audit protocol has been previously published by the authors and is described elsewhere [55]. Briefly, all incoming deliveries were weighed, photographed, and annotated with the donor’s name, delivery date, type of food, product information (brand and product name, variety), weight (kilograms (kg)), and date-marking details. Data pertaining to 1054 kg (1% of 86,050 kg) of food were excluded from the nutrition classification as information was either missing (e.g., product with no labeling) or unattainable (product arrived in a frozen mixed load and was excluded as sorting and data collection time caused it to be considered a food safety risk) [55]. The remaining data were classified according to diet, food, and nutrient criteria.

*Diet-based classification* assessed consistency with the Australian Dietary Guidelines (ADGs) and categorized each food as either healthy, that is, belonging to one of the ‘nutritious’ five food groups (FFGs) (e.g., grains and cereals; vegetables and legumes; fruit; meat and alternatives; dairy and alternatives) and water) or unhealthy (i.e., energy-dense, nutrient-poor ‘discretionary’ foods) [56]. Ingredients lists were used to assist in the categorization of mixed food products, with the characterizing ingredient used to determine the food’s classification. If the predominant ingredient was added sugar, fat, and/or salt, the food was classified as a discretionary food. Consistent with other NCS research, the ingredients list and purpose of the food were the basis of consensus decisions [44].

*Food-based classification* employed the NOVA system which categorizes foods into four groups based on the nature, extent, and purpose of industrial processing: Group 1 (unprocessed), Group 2 (processed culinary ingredient), Group 3 (processed), or Group 4 (ultra-processed) [52]. Ultra-processed foods (UPFs) can be identified based on the presence of cosmetic additives (e.g., colors, flavors, and texture enhancers such as emulsifiers) in the ingredient list, known as markers of ultra-processing (MUPs) [57,58]. Consumption of UPFs is associated with poor health and the development of chronic disease [52], and thus they were categorized as unhealthy. Some preservatives, particularly in canned foods (e.g., citric acid), are not categorized as MUPs as defined by previous research [59], and this was applied in the current study. All other groups were categorized as healthy.

The *nutrient-based classification* utilized the HERNGs. The HERNGs focus on three nutrients of concern: saturated fat, salt, and added sugars (consistent with US and Australian food labeling deliberations) [60,61]. Different thresholds for each nutrient for nine of the eleven food group categories are specified: (1) fruits and vegetables; (2) grains; (3) protein; (4) dairy; (5) nondairy alternatives; (6) beverages; (7) mixed dishes; (8) processed and packaged snacks; and (9) desserts. The remaining categories, condiments and cooking staples and miscellaneous items, were not assessed, but are included in the total weight of food distributed. Thresholds are based on nutrients per single food serving as declared on the nutrition facts label. Foods compared with thresholds are ranked in three tiers (‘choose often’, ‘choose sometimes’, and ‘choose rarely’) across nine food categories. In Australia, nutrients per serving size and per 100 g are required on the nutrition information panel, but declared serving sizes are not standardized; therefore, the amount per 100 g is typically used [62]. In this study, the recommended serving sizes (SSs) specified in the ADGs’ Australian Guide to Healthy Eating [63] were used, and if not specified, for example in ready meals, the SS was based on evidence from the literature [64,65] or a review of the SS within the product category, and a ‘typical’ SS was determined. Additional detail is provided in the Supplementary Materials.

### 2.1. Suitable versus Unsuitable Food

The categorizations within each NCS were grouped together into a binary output [healthy/suitable or unhealthy/unsuitable] for analysis. The ADGs’ binary classification based on ‘suitable’ FFG foods and ‘unsuitable’ discretionary food was applied. The approach previously applied to NOVA by Dickie et al. (2022) [44] was used, with ‘suitable’ products in Groups 1–3 and ‘unsuitable’ referring to those in Group 4. For the ternary HERNGs classification [13], ‘suitable’ included ‘choose often’ and ‘unsuitable’ included the ‘choose sometimes’ and ‘choose rarely’ foods. Cooking staples and condiments are not ranked under the HERNGs but are considered ‘suitable’ given they encourage home cooking and meal preparation [51], so they were included in the ‘suitable’ category for this study.

### 2.2. Statistical Analysis

All audit data were initially entered into Microsoft Excel (Version 2019, Redmond, WA, USA), and descriptive statistics were generated related to the type of product and frequency of donation. Products were also classified according to each NCS, and then subsequently grouped together into a binary output (suitable and unsuitable) under each NCS for further analysis. Using IBM SPSS Statistics (Version 29), the Cohen κ coefficient was calculated to determine the percentage agreement between each NCS pairing. The κ coefficient was interpreted using values from Landis and Koch, where <0 indicates no agreement, 0–0.20 is slight, 0.21–0.40 is fair, 0.41–0.60 is moderate, 0.61–0.80 is substantial, and 0.81–1 is almost perfect agreement [66]. The level of agreement between the NCSs was further analyzed according to food group, with the scientific underpinnings and methodological features of each NCS considered to explain any similarities and differences in agreement across and within food groups.

## 3. Results

Seventy-four donations were received by FBWA over five consecutive days with 1500 images documenting each donation and two loads of procured food sourced and purchased by FBWA. The total weight of all food was 108,509 kg, consisting of 1225 products. Donations accounted for 79% (86,050 kg) of the total weight of food received and 99% (n = 1221) of all products. Procured loads and 1% by weight (1054 kg) of donations were excluded from the analysis for reasons described previously. A total of 1216 products (84,996 kg) from 72 donations, as depicted in Table 1, were analyzed.

Supermarkets contributed almost 40% (n = 27/72) of all donations and 70% (n = 855/1216) of all products received, while intraorganizational donations (national and local donations from within FBWA’s network) comprised the smallest number of donations and products overall (3%, n = 2/72 and 1%, n = 11/1216, respectively).

**Figure 1:**
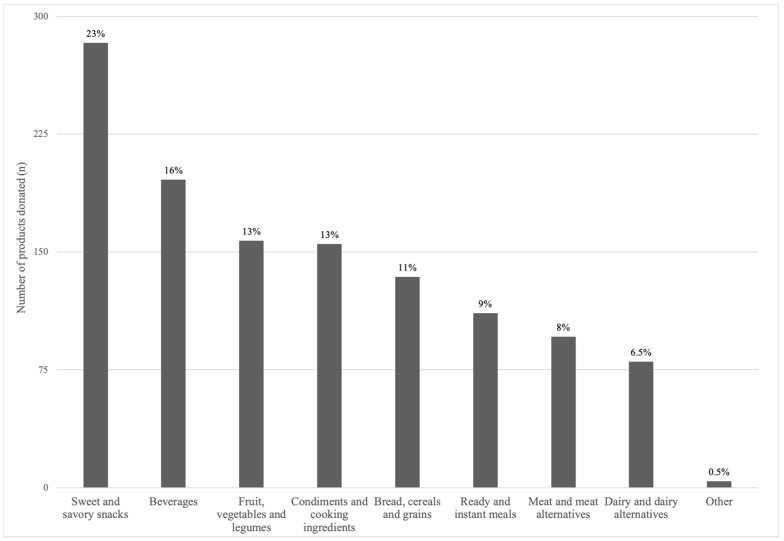
Number of donated products (total = 1216) by food group donated to FBWA over a 5-day audit.

The types of products donated spanned nine different food groups, from sweet and savory snacks to vegetables, legumes, and fruit, as illustrated in Figure 1.

### 3.1. Types of Food and Beverages Donated

Sweet and savory snacks accounted for 23% (n = 283) of all donations and included confectionary, chocolate, sweet biscuits, pastries, cake mixes, ice cream, jelly, snack bars, crisps, jerky, and savory crackers. Beverages comprised 16% (n = 196) of all donations, of which 40% were sugar-sweetened beverages (SSBs) (e.g., soft drinks (soda), cordial, and fruit and sports drinks); 19% water (plain, still, and sparkling); 19% coffee (ground, instant, and pods), tea, flavored coffee sachets, and drinking chocolate; 13% artificially sweetened beverages; and 9% fruit and vegetable juices. Vegetables (n = 79), fruit (n = 39), and legumes (n = 39) comprised 13% of products received, of which 56% were canned, 39% fresh, 1% frozen, and 4% dried. Condiments (n = 109), cooking ingredients (n = 39), and oils (n = 8) accounted for 13% of overall donations. Condiments included tomato sauce, mustard, chutney, and mayonnaise, pickled/brined products (e.g., jalapenos, olives), spreads (e.g., jam, nut butters), sauces (e.g., soy sauce, pasta sauce), stock, and seasonings (e.g., herbs and spices). Cooking ingredients included flours, sugars, and cocoa. Breads, cereals, and grains accounted for 11% (n = 134) of all donations, of which 38% were breakfast cereals, 36% grains (e.g., pasta, rice), 16% bread, and 10% crispbreads. Instant and ready meals accounted for 9% (n = 111), with canned soup or instant soup mixes and noodles comprising 52%, chilled ready meals 42%, and meal kits 6%. Meat and meat alternatives comprised 8% (n = 96) of all donations, of which 44% was fresh meat (plain, marinated, crumbed), 18% processed meat, 3% prepared meat products (e.g., chicken Kiev, ready pulled pork), 13% tinned fish, 10% nuts and seeds, and 6% each of plant-based meat and eggs. Dairy and alternatives comprised 7% (n = 80) of all donations, of which 50% was fresh/long-life milk (n = 40), including plant-based varieties, 25% yoghurt, 11% cheese, and 14% other dairy-based products (e.g., liquid breakfast drinks). Lastly, other foods (n = 4) comprised baby food pouches.

The types of food products (total number and weight) received by donors are outlined in Table 2a,b.

### 3.2. Donors

Supermarkets donated the greatest number of products across all food groups by product type (e.g., 88% of all beverages, 90% of SSBs, 92% of all water, 82% of sweet and savory snacks, 69% of condiments and cooking ingredients) and for over half of all food groups (e.g., 93% of all instant and ready meals, 67% of beverages, and 53% of all sweet and savory snacks) by total weight. Vegetables, legumes, and fruit were supermarkets’ least likely food group to donate, by both weight and product count. Growers and producers, meal delivery companies, and the public were more likely to donate these products. Supermarkets and the public tended to donate canned varieties, while meal delivery companies were more likely to donate fresh produce. Condiments and cooking ingredients were largely donated by supermarkets (68% of products) and transport companies (2% of total products), accounting for 96% by weight.

Supermarkets donated 70% (by product count) of ready/instant meals (e.g., chilled ready meals, canned soups, and instant noodles) and 93% of this food group type by weight. Two thirds of all bread, cereal, and grain products, such as breakfast cereals and other grains (e.g., pasta and rice), were donated by supermarkets, but this only equated to 15% by weight. Both manufacturers and transport companies donated fewer bread and cereal products, but these accounted for 77% of the total weight. Manufacturers largely donated bread.

Supermarkets donated 61% of all meat and meat alternative products (e.g., fresh and processed meat, and, to a lesser extent, tinned fish, nuts, and seeds), which accounted for one third of the weight of this food group. Similarly, 66% of all dairy and dairy alternatives were from supermarkets, which also equated to just over a third of the weight of this food type. Manufacturers donated 14% of dairy foods (52% by weight), with milk and yoghurt being most common. Supermarkets were responsible for all the ‘other’ food donations.

### 3.3. Nutrition Categorization

**Figure 2:**
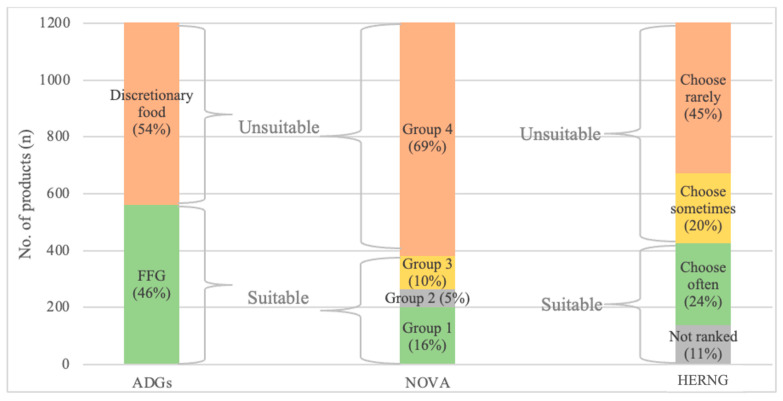
Nutrition classification of the total number of products donated to FBWA over 5 days by Australian Dietary Guidelines (ADGs), NOVA, and Healthy Eating Research Nutrition Guidelines (HERNGs).

Figure 2 shows the proportion of donated products classified under the three NCSs (the ADGs, NOVA, and the HERNGs).

The ADGs’ method categorized 54% (n = 654 products; 40,848 kg) of all donated food as discretionary and 46% (n = 562; 44,148 kg) as FFG foods. NOVA categorized 69% (n = 834 products; 33% by weight) as ultra-processed (Group 4). The HERNGs categorized 45% (n = 544; 33,643 kg) of donated food as ‘choose rarely’ and 24% as ‘choose often’ (n = 289; 20,769 kg).

### 3.4. Comparison of NCSs

**Figure 3:**
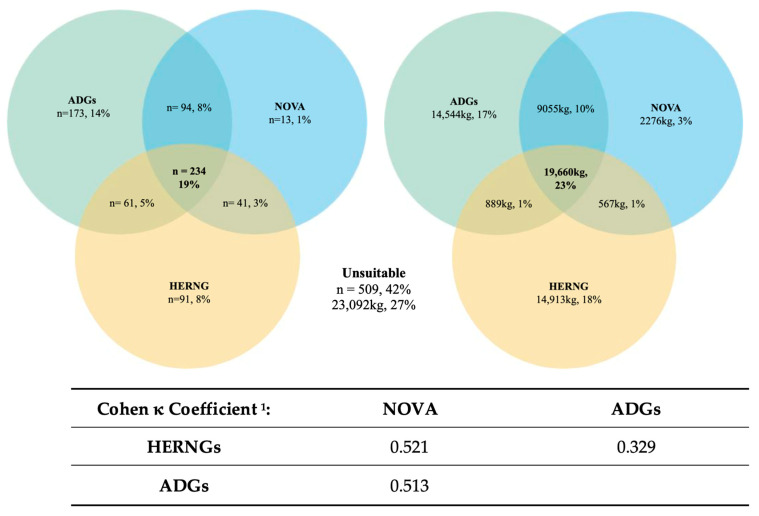
Agreement between each nutrition classification scheme ((NCS), either suitable or unsuitable binary output) using the number of products, weight, and the calculated level of agreement of the number of products using Cohen κ coefficient ^1^. ^1^ κ coefficient interpreted using values from Landis and Koch [66]: <0.0 = no agreement; 0–0.20 = slight; 0.21–0.40 = fair; 0.41–0.60 = moderate; 0.61–0.80 = substantial; 0.81–1 = almost perfect.

NCSs were further categorized as a binary output of suitable or unsuitable (Table 3). ‘Suitable’ for the ADGs comprised FFG, for NOVA it comprised Groups 1, 2, and 3, and for the HERNGs it comprised ‘choose often’ and ‘not ranked.’ For the ADGs, ‘unsuitable’ comprised discretionary foods, for NOVA it comprised Group 4, and for the HERNGs it comprised ‘choose sometimes’ or ‘rarely’. The Venn diagrams (Figure 3) illustrate the total number and weight of suitable products as categorized by each NCS and their intersections.

**Figure A1:**
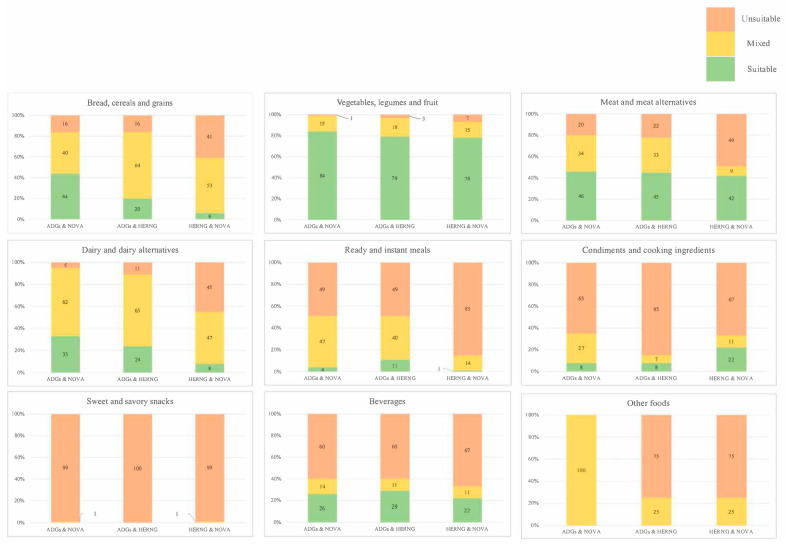
Percentage agreement (suitable, mixed, and unsuitable) by food group for each nutrition categorization scheme (Australian Dietary Guidelines (ADGs), NOVA, and Healthy Eating Research Nutrition Guidelines (HERNGs)) grouping.

The NCSs agreed that 19% (n = 234) of all donated products were suitable, with the ADGs and NOVA having greater agreement than the ADGs and HERNGs or the HERNGs and NOVA (n = 94, 61, and 41, respectively). The ADGs categorized more products as suitable than the HERNGs or NOVA (n = 173, 91, and 13, respectively). By weight, this equates to 23% (19,660 kg) of suitable products, with the ADGs and NOVA agreeing on 9055 kg, the ADGs and HERNGs on 889 kg, and the HERNGs and NOVA on 567 kg. The HERNGs rated the most donations suitable by weight compared with the ADGs and NOVA (14,913 kg, 14,544 kg, and 2276 kg, respectively). The remaining 23,092 kg (509 products), as shown in Figure 3, was rated ‘unsuitable’. Agreement between each NCS using Cohen’s κ coefficient found that NOVA and the HERNGs (κ = 0.521) had moderate agreement, as did the ADGs and NOVA (κ = 0.513), and the ADGs and HERNGs had fair agreement (κ = 0.329). The agreement between each NCS by food product category allowed further exploration of the suitability across and within food groups, as illustrated in Table A1 and Figure A1 (Appendix A) and explained below.

Overall, the food categories with the highest agreement, that is, ranked similarly as either suitable or unsuitable across the NCSs, were sweet and savory snacks, vegetables, legumes and fruit, beverages, and meat and meat alternatives. Low agreement was evident in dairy and dairy alternatives, bread, cereals, and grains, ready meals, and condiments and cooking ingredients.

### 3.5. Bread, Cereals, and Grains

There was unanimous agreement that sugar-sweetened breakfast cereals were ‘unsuitable’ by all NCSs. Under ADGs and NOVA categorization, 44% (n = 59) of breads, cereals, and grains were considered suitable, including all plain varieties of oats, rice, pasta, and noodles. Of these products, the HERNGs only considered oats and brown rice ‘suitable’ as all non-wholegrain varieties were categorized as ‘unsuitable’ using this classification.

There was a high level of disagreement between the ADGs and HERNGs (as per the Cohen κ coefficient results in Figure 3), with 64% (n = 85) of products deemed ‘unsuitable’ by the latter scheme. This was largely due to the HERNGs categorizing non-wholegrain varieties of pasta, bread, wraps, and crumpets above a sodium threshold, breakfast cereals with dried fruit outside the sugar criteria, and wholegrain crispbreads as ‘unsuitable’. Products like wheat biscuit cereals were ‘suitable’ under the HERNGs and ADGs, but ‘unsuitable’ according to NOVA due to the presence of malt extract, an industrially derived sweetener. NOVA also deemed commercially produced breads and most other breakfast cereals ‘unsuitable’ due to the presence of modified starches and/or industrially derived sweeteners.

### 3.6. Vegetables, Legumes, and Fruit

Vegetables, legumes, and fruit yielded 78 to 84% agreement on suitability for the ADGs and NOVA, the ADGs and HERNGs, and the HERNGs and NOVA. The misalignment concerned baked beans, creamed corn, and tinned beetroot. NOVA deemed these products ‘unsuitable’ due to the presence of MUPs such as thickeners, flavors, and high-fructose corn syrup, although this was not consistent across all brands. Similarly, these products were often too high in sodium or sugar according to the HERNGs, and therefore ‘unsuitable’, but were ‘suitable’ under the ADGs. Tinned fruit in syrup was only considered ‘suitable’ under NOVA, yet it was considered ‘unsuitable’ by the ADGs and NOVA, although there were two exceptions which contained MUPs.

### 3.7. Meat and Meat Alternatives

Meat and meat alternatives (e.g., plain non-marinated meats, tinned fish, eggs, nuts, and seeds) had 42 to 45% alignment for the ADGs and NOVA (46%), the ADGs and HERNGs (45%), and the HERNGs and NOVA (42%). This included plain non-marinated meats, tinned fish, eggs, nuts, and seeds. Likewise, processed meats (e.g., salami, pepperoni, chorizo) were deemed ‘unsuitable’ by all schemes, but the HERNGs and NOVA also categorized marinated and prepared meats, plant-based products, and some varieties of flavored tinned fish as ‘unsuitable’. Under NOVA this was due to the presence of emulsifiers (e.g., in marinades) and/or flavor extracts, and for the HERNGs these products were considered too high in sodium and/or saturated fat. It was these products that were the source of misalignment between the ADGs and NOVA (n = 31) and the ADGs and HERNGs (n = 32).

### 3.8. Dairy and Dairy Alternatives

There were many inconsistencies within this category. The ADGs and NOVA categorized 33% (n = 26) of this category as ‘suitable’, comprising all fresh or long-life, full- or low-fat, unflavored cow’s milk and yoghurt. Under the HERNGs, however, all plain, full-fat varieties of milk and yoghurt were ‘unsuitable’ due to exceeding the saturated fat threshold. High-fat cheeses (e.g., brie, camembert) were also considered ‘unsuitable’ according to the HERNGs and the ADGs as well, but were suitable under NOVA. Flavored and sweetened milk and yoghurts and plant-based milks were deemed ‘unsuitable’ under NOVA due to the presence of cosmetic additives (e.g., flavor, color), gelling or thickening agents, and/or industrially derived sweeteners. Plant-based milks were the source of misalignment between NOVA (‘unsuitable’) and the other NCSs (‘suitable’). Plant-based cheese and liquid breakfast drinks were deemed ‘unsuitable’ by the HERNGs and NOVA but considered ‘suitable’ by the ADGs. There were only four products that were deemed universally ‘unsuitable’, including cream cheese and crumbed camembert wedges.

### 3.9. Ready and Instant Meals

There was a high level of agreement between NOVA and the HERNGs, with 85% (n = 95) of ready and instant meals deemed ‘unsuitable’. This included savory pastry-based products like quiches, pies, and samosas, as well as pizzas, creamy pasta dishes, instant noodles, and packet soups. These types of foods contain gelling/thickening agents, flavors, colors, and sweeteners, and high levels of sodium and/or saturated fat. The ADGs aligned with NOVA and the HERNGs regarding these foods; however, there was disagreement between the ADGs and the other NCSs concerning tinned spaghetti and certain types of canned soups (e.g., vegetable-based varieties), which were deemed ‘suitable’ by the ADGs but ‘unsuitable’ by the others. Ready meals (e.g., meat- and rice-based dishes) typically contained flavors, colors, and thickeners, as well as being high in sodium and/or saturated fat, so they were deemed unsuitable by NOVA and the HERNGs, respectively, but ‘suitable’ by the ADGs given that the meals’ characterizing ingredients were carbohydrate or protein sources. All Mexican-style meal kits (e.g., tacos, burritos) were collectively considered ‘unsuitable’, but salad-based kits were ‘suitable’ according to the ADGs, as these products were predominantly vegetables.

### 3.10. Sweet and Savory Snacks

There was a high level of agreement between the three NCSs within this category. The ADGs and HERNGs had 100% agreement that these foods were ‘unsuitable’. NOVA was also aligned with the other two NCSs for 98% of products, but the remaining 2% of foods, like beef jerky and salted popcorn, were the source of the incongruities as they were considered ‘suitable’ under NOVA, yet ‘unsuitable’ according to the ADGs and HERNGs.

### 3.11. Condiments and Cooking Ingredients

Under the HERNGs, condiments like sauces and savory condiments were considered ‘suitable’ because they promote home cooking and meal preparation [51], but this was at odds with NOVA and the ADGs, and was the main source of misalignment between the schemes in this product category. All three schemes, though, considered tomato sauce, condensed milk, and chocolate spread as ‘unsuitable’. Likewise, cooking ingredients such as flour, spices, and tomato paste were deemed ‘suitable’ according to all NCSs. Items like salt, sugar, and honey were considered ‘suitable’ under NOVA and the HERNGs, but the ADGs deemed these products ‘unsuitable’.

### 3.12. Beverages

It was unanimous across all three NCSs that soft drinks (sugar-sweetened and intensely sweetened), sports drinks, fruit drinks, energy drinks, iced teas, and flavored coffee drinks were ‘unsuitable’, while all schemes considered water (plain or unflavored sparkling varieties) to be ‘suitable’. Under both the ADGs and NOVA, 100% fruit juices were also ‘suitable’, but there were some brands of long-life fruit juices that contained added flavors and colors, which relegated these to ‘unsuitable’ under NOVA. The sugar content of all fruit juices surpassed the HERNGs’ sugar criteria. Plain varieties of coffee and tea were unanimously considered ‘suitable’, except for instant coffee, which was considered ultra-processed, as were flavored tea varieties [67]. Likewise, flavored sparkling mineral waters were ‘suitable’ according to the ADGs and HERNGs, but ‘unsuitable’ under NOVA.

### 3.13. Other Foods

This category consisted of baby food pouches, which typically contained gelling agents (MUPs), and three of the four products exceeded the sugar criteria under the HERNGs and so were ‘unsuitable’ according to NOVA and the HERNGs, but all were ‘suitable’ according to the ADGs.

## 4. Discussion

This study explored the nutritional quality of food donated to an Australian food bank over a five-day period using diet-, food-, and nutrient-based classification methods. The results represent an important contribution to the literature given a comprehensive analysis of a food bank inventory has not been conducted in Australia previously.

A total of 42% of all donated food products received (27% by weight) were consistently deemed unsuitable based on their nutritional quality. These products were either discretionary food or ultra-processed food according to the ADGs and NOVA, respectively, and are not recommended as part of a healthy diet [56]. While it is difficult to compare these results with other food bank inventory studies due to differences in methodology and outcome measures, the proportion of donated, nutritionally unsuitable food appears to be higher in this current study compared with US research which found 25% of total products received [8] or 8 to 19% [42,45] of the total weight of the inventory to be unsuitable. The distinction between the total number and total volume of products received must be emphasized when assessing the appropriateness of donated food. While the total number of products reflects the range or types of items donated, from which quality can be gauged, it does not reflect the proportional weight contributed by each product. Products donated ranged from 380 g packets of potato crisps to industrial quantities of the savory snack (e.g., 129 kg); therefore, this must be taken into account when assessing the nutritional quality of the food supply. The universal measure of food banking success is currently a weight-based metric that may indicate the overall volume of products received; however, it does not reflect the quality of the food. In isolation, both the total product and total volume measures lack context and specificity. This underscores the need to incorporate measures that capture *both* the quantity and the quality of food to effectively assess the nutritional value and dietary contribution of donations. This echoes previous calls to reform the existing weight-based metric [13,23].

The findings of the current study are consistent with existing literature that suggests that food banks are unable to support the provision of healthy food to clients [20], as only 19% of products (23% by weight) were deemed collectively suitable by all NCSs for distribution. While the symbolic value of food beyond its nutrient content [68,69] must be acknowledged, clients accessing food banks do not desire discretionary food and place less value on these products than healthier foods [25,40,41]. Charitable food recipients in a WA study noted the abundance of unhealthy food on offer: ‘*…there was a lot of junk food…I was disappointed*’ [70]. The redistribution of unhealthy food may also increase the health risks and exacerbate existing medical conditions of this already vulnerable population subgroup who experience disproportionately high rates of diet-related chronic diseases [35,68]. Food banking clients across the globe have expressed difficulty in managing their chronic conditions with the food received [30,70,71]. In effect, food banks may be harming the very people they are intending to help [68]. This is not a criticism of food banks per se; they provide an essential service to clients experiencing food insecurity. Rather, the findings reflect the position of food banks as ‘small actors in a much larger and unwieldly food system’ [72] (p. 54), where the food industry, particularly supermarkets, exercises incredible power and influence [73]. This is especially true for FBWA, who rely almost entirely on the food industry for donations.

Supermarkets were responsible for 70% of all types of products (40% by weight) received. From a product variety perspective, supermarkets donated over two thirds of all dairy, meat, and cereal products. Supermarkets also donated the widest variety of sweet and savory snacks and beverages, and almost all SSB products. In contrast, less than half of the fruit, vegetable, and legume product donations were attributable to supermarkets. Supermarkets have declared their intention to help feed the hungry and reduce food waste (food which has been removed from the food system, but is still fit for consumption [74]) as part of their corporate social responsibility statements [75]. Despite their noble intentions, food donations are not simply an altruistic gesture [76,77]. Rather, supermarkets are driven by economic considerations [76,77], and concerned with ‘ridding their systems and locations of unwanted or surplus goods’ [78] to ensure zero waste with regard to their inventories [15]. Meanwhile, food banks are increasingly committed to the distribution of healthier food to clients and prioritize quality over quantity [18,78]. The misalignment in values between supermarkets and food banks is evident, which creates tension and undermines the ability of food banks to effectively serve their clients [77,78]. While supermarkets bask in the glow of their purported socially responsible behaviors, and avoid costly waste disposal fees, food banks, despite their best intentions, contend with retail leftovers and struggle to provide appropriate and nutritious food for clients.

The charitable nature of food banking makes it difficult to ‘question the giver of the gift’ [72] (p. 50), especially when there is a perceived power imbalance between giver and receiver [77,78]. Food banks report feeling compelled to accept donations of any quality for fear of fracturing relationships with donors and the potential implications for supply [18,78] as well as feeling uncomfortable with surplus food ending up in landfill [38]. However, food banks have a duty of care, a moral responsibility, and a legal obligation to their clients under the Right to Food [79] to ensure the quality of food distributed. As a manager in the US stated, ‘*…we don’t serve corporations, we don’t serve food manufacturers. We serve people who are hungry and who need healthy food…*’ [68] (p. 52). Indeed, there are examples of food banks in the US that have inverted the power dynamic and have ceased to accept unhealthy food through the initiation of nutrition policies [68]. The Food Bank of New York City established the ‘No Soda, No Candy’ policy in 2004 in an effort to improve the quality of food distributed and to better meet clients’ needs [80]. The rejection of unhealthy food is a way for food banks to demonstrate ‘client-centered’ food banking [81], which assists recipients to eat better and become healthier [68]. Progress concerning nutrition policy at Australian food banks and the CFS more broadly is limited [82], and this constitutes a policy gap [83]. Only one Australian jurisdiction has developed specific nutrition guidelines for the CFS [84], but the utility of the guidelines and evidence of implementation remain unknown, especially the stipulated proportional volume allowances that accompany the guidelines. As an initial step, Australian food banks should cease to accept the donation of discretionary, ultra-processed food like soft drinks and sweet and savory snacks.

Central to the initiation of nutrition policy is the requirement for an NCS to determine the suitability of donated food. The results of this research showed that 23% of donated food by weight (19% of all products) was considered ‘suitable’ by all NCSs and this included wholegrains, fresh fruit and vegetables, plain meat, low-fat dairy products, and water. A total of 27% by weight (42% of all products) was collectively deemed ‘unsuitable’, and this included SSBs and sweet and savory snacks. The remaining 50% of products by weight (39% of all products received) had no agreement. These results reveal a lack of alignment between each scheme due to conceptual (nutrient-, food-, or diet-based) and technical differences (e.g., nutrients and/or ingredients considered, thresholds used, binary categories) [44]. The application of the three NCSs provides insight into the practicality, feasibility, and therefore suitability of each scheme for use in monitoring and assessing the nutritional quality of donated food. The NCS anomalies across and within certain food groups concern many products which play a central role in the diet and are important commodities (e.g., bread, breakfast cereals, ready meals) for food banks. This raises the question about the suitability of these NCSs for use at Australian food banks and within the wider CFS. NOVA is increasingly being used by policymakers to underpin public health decisions [85] due to the strong association between UPFs and the development of diet-related chronic disease [57]. For food banks, however, food processing is an important consideration given it delays perishability, ensures safety, contributes to palatability, and can simplify meal preparation [85,86]. A recent European prospective cohort study found that UPFs like commercial bread and cereals were *inversely* associated with the risk of multimorbidity, while ready meals were not associated with risk of multimorbidity [87]; these are important findings to consider within the context of the CFS. In its current form, NOVA appears too idealistic and uncompromising as an NCS for food banks. While the ADGs classified the highest number of products as suitable, the binary categorization masks the many grey areas of food groupings [13,44]. It is within this undefined space that many products important to food banks would qualify (e.g., ready meals), and because the relative healthfulness of these items is not easily determined [88], this renders the ADGs challenging to apply. The HERNGs are complex to interpret, with three different thresholds for three distinct nutrients across nine specific categories. Despite their consensus status in the US, their application and utility in a busy food bank seem problematic without digitalization and/or extensive volunteer training. The recognition of the importance of condiments and cooking ingredients is commendable and a key aspirational quality of the HERNGs is their emphasis on wholegrains. However, this diminishes the important roles white varieties of bread, cereal, and grains occupy in the diet, and the value these products likely represent to food bank clients. Lastly, the focus on nutrients is reductionist in its approach [59,88].

The lack of agreement between the schemes demonstrates tension, and in their current forms, for varied reasons, all three are impractical for implementation at Australian food banks. Most importantly, none of the systems collectively consider the specific context of the food relief sector, such as the importance of providing clients with food and cooking ingredients to aid meal construction [25,89], the spectrum of clients’ food preparation facilities, or the unique operational characteristics of food banks.

Overall, the results confirm the poor nutritional quality of food donated to Australian food banks and the need to improve the nutritional quality of donated food in Australia, echoing previous calls for nutrition-focused food banking [90,91]. There is also a need to develop and test a fit-for-purpose NCS for food donated to food banks that fosters transparency and accountability among donors by regulating the types of food that can be donated. As an initial step toward the development of a comprehensive nutrition policy, and given the unanimous findings across NCSs, the donation of discretionary, ultra-processed food (e.g., SSBs, sweet and savory snacks) should be prohibited. Reforming the current weight-based metric to incorporate quantity *and* quality components would underpin and aid the implementation of nutrition-focused initiatives and foster a reorientation of the food banking system toward clients rather than supermarkets.

## 5. Conclusions

This study generated important insight into the nutritional quality of donated food at an Australian food bank. The application of the three NCSs provides insight into the practicality, feasibility, and suitability of each scheme for use in monitoring and assessing the nutritional quality of donated food. The results revealed a need to reform the food banking system, especially in the context of rising demand, to ensure the provision of appropriate and nutritious food to clients experiencing food insecurity. There is a clear requirement for ongoing monitoring, evaluation, and research into the quality of donated food received and the food system supporting this in the global and local context. Research is needed to support the development of specific nutrition guidelines for Australian food banks to ensure suitable food is available to clients.

## 6. Strengths and Limitations

This research is the first comprehensive assessment of the nutritional quality of donated food at Australian food banks and represents an important contribution to the literature. While the results reflect a once-off 5-day audit, they are likely to be relevant to other food banks in Australia and the CFS more broadly. Ongoing monitoring is recommended to help guide operational decisions and inform policy. Indeed, FBWA are positioning themselves to undertake routine audits to assess nutritional quality, and the development of an NCS to inform nutrition policy and the development of guidelines would support these efforts. The collaboration between FBWA and the researchers must be emphasized, particularly FBWA’s support, willingness, and engagement with this research.

## Figures and Tables

**Table 1:** Total number of donations and products received by FBWA over a 5-day audit by donor type.

Type of Donor	Total No. Donations Received (%)	Total No. Products Received (%)	Total Weight (kg) of Products Received (%)
Supermarkets	27 (37)	855 (70)	34,090 (40)
Food manufacturers	11 (15)	45 (4)	13,302 (16)
Transport logistics and distribution	9 (13)	19 (1.5)	17,044 (20)
General public	9 (13)	189 (16)	921 (1)
Growers and producers	8 (11)	15 (1)	6300 (7)
Meal delivery companies	3 (4)	64 (5)	6698 (8)
Other retail businesses	3 (4)	18 (1.5)	1507 (2)
Intraorganizational	2 (3)	11 (1)	5134 (6)
**TOTAL**	**72 donations (100)**	**1216 products (100)**	**84,996 (100)**

**Table 2:** (**a**) Total number (n) of donated products by food group and donor type. (**b**) Weight (kg) of donated products by food group and donor type.

	Sweet and Savory Snacks kg (%)	Beverages kg (%)	Vegetables, Legumes, and Fruit kg (%)	Condiments and Cooking Ingredients kg (%)	Bread, Cereals, and Grains kg (%)	Ready and Instant Meals kg (%)	Meat and Meat Alternatives kg (%)	Dairy and Dairy Alternatives kg (%)	Other Foods kg (%)	Total Weight of Products kg (%) per Donor
(**a**)										
Supermarkets	231 (82)	173 (88)	68 (43)	106 (68)	83 (62)	78 (70)	59 (62)	53 (66)	4 (100)	**855 (70)**
General public	25 (9)	16 (8)	41 (26)	37 (24)	35 (26)	19 (17)	9 (9)	7 (9)	-	**189 (16)**
Meal delivery companies	-	-	25 (16)	8 (5)	4 (3)	10 (9)	9 (9)	8 (10)	-	**64 (5)**
Manufacturers	9 (3)	-	3 (2)	1 (1)	9 (7)	2 (2)	10 (11)	11 (14)	-	**45 (4)**
Transport, logistics, and distribution	4 (1)	4 (2)	2 (1)	3 (2)	3 (2)	1 (1)	1 (1)	1 (1)	-	**19 (2)**
Growers and producers	-	-	8 (5)	-	-	-	7 (7)	-	-	**15 (1)**
Intraorganizational	-	-	10 (7)	-	-	-	1 (1)	-	-	**11 (1)**
Other retail	14 (5)	3 (2)	-	-	-	1 (1)	-	-	-	**18 (1)**
**Total n (%)**	**283 (23)**	**196 (16)**	**157 (13)**	**155 (13)**	**134 (11)**	**111 (9)**	**96 (8)**	**80 (6.5)**	**4 (0.5)**	**1216 (100)**
(**b**)										
Supermarkets	7708 (53)	3222 (67)	886 (7)	7618 (47)	628 (15)	5455 (93)	3987 (31)	4581 (33)	5 (100)	34,090 (40)
General public	21 (0)	53 (1)	422 (3)	65 (0)	256 (6)	18 (0)	8 (0)	78 (1)	-	921 (1)
Meal delivery companies	-	-	2599 (21)	426 (3)	109 (2)	96 (2)	1909 (15)	1559 (11)	-	6698 (8)
Manufacturers	3765 (26)	-	137 (1)	185 (1)	1017 (23)	32 (1)	810 (6)	7356 (52)	-	13,302 (16)
Transport, logistics, and distribution	1462 (10)	1535 (32)	1806 (15)	7861 (49)	2377 (54)	225 (4)	1275 (1)	503 (4)	-	17,044 (20)
Growers and producers	-	-	2785 (22)	-	-	-	3515 (27)	-	-	6300 (7)
Intraorganizational	-	-	3790 (31)	-	-	-	1344 (10)	-	-	5134 (6)
Other retail	1456 (10)	17 (0)	-	-	-	34 (1)	-	-	-	1507 (2)
**Total kg (%)**	**14,412 (17)**	**4826 (6)**	**12,425 (15)**	**16,156 (19)**	**4387 (5)**	**5859 (7)**	**12,849 (15)**	**14,077 (17)**	**5 (0)**	**84,996 (100)**

**Table 3:** Summary of nutrition classification scheme (NCS) categorization into suitable/unsuitable groupings.

	Suitable	Unsuitable
**ADGs**	FFG	Discretionary food
**NOVA**	Group 1 Group 2 Group 3	Group 4
**HERNGs**	Choose often Not ranked	Choose sometimes Choose rarely

## Data Availability

The data compiled as a result of the current study are available from the corresponding author upon reasonable request.

## Supplementary Materials

The following supporting information can be downloaded at https://www.mdpi.com/article/10.3390/nu16040509/s1, Table S1. Serve size information for categorization according to the Healthy Eating Nutrition Research Guidelines for the Charitable Food System. References [64,65,92] are cited in the supplementary materials.

## Appendix A

**Table A1:** Percentage agreement (suitable (S), unsuitable (U), and total (T)) by food group for each nutrition categorization scheme (Australian Dietary Guidelines (ADGs), NOVA, and Healthy Eating Research Nutrition Guidelines (HERNGs)) grouping.

Food Group																												
		Bread, Cereals, and Grains (n = 134)			Vegetables, Legumes, and Fruit (n = 125)			Meat and Alternatives (n = 96)			Dairy and Alternatives (n = 80)			Ready and Instant Meals (n = 111)			Sweet and Savory Snacks (n = 283)			Condiments and Cooking Ingredients (n = 156)			Beverages (n = 196)			Other Foods (n = 4)		
		S	U	**T**	S	U	**T**	S	U	**T**	S	U	**T**	S	U	**T**	S	U	**T**	S	U	**T**	S	U	**T**	S	U	**T**
		%	%	**%**	%	%	**%**	%	%	**%**	%	%	**%**	%	%	**%**	%	%	**%**	%	%	**%**	%	%	**%**	%	%	**%**
**NCSs**	**ADGs and NOVA**	44	16	**60**	84	1	**85**	46	20	**66**	33	5	**38**	4	49	**54**	1	99	**100**	8	65	**73**	26	60	**86**	0	0	**0**
	**ADGs and** **HERNGs**	20	16	**36**	79	3	**82**	45	22	**67**	24	11	**35**	11	49	**60**	0	100	**100**	8	85	**93**	29	60	**89**	0	75	**75**
	**HERNGs and NOVA**	6	41	**47**	78	7	**85**	42	49	**91**	8	45	**53**	1	85	**87**	1	99	**100**	22	67	**89**	22	67	**89**	0	75	**75**
