# Supplementary material for: The Nutritional Quality of Food Donated to a Western Australian Food Bank
Source: Nutrients. 2024 Feb 11;16(4):509. doi: 10.3390/nu16040509 (PMC10891512; doi:10.3390/nu16040509)
Supplement: Supplementary file 1 [file nutrients-16-00509-s001.zip › nutrients-2799417-supplementary.pdf]

Supplementary Materials

1. Supplementary Table S1. *Serve size information for categorization according to the Healthy Eating Nutrition Research Guidelines for the Charitable Food System*

Supplementary Table S1 – Serve size (SS) information used for categorization of food according to the Healthy Eating Nutrition Research Guidelines for the Charitable Food System*

Food item	Serve size	Source	HENRG food category classification†
**Dairy**			
Liquid breakfast drinks	250mL	Review of product category SS	Dairy
Yoghurt, all types (incl. flavoured)	200g	ADG	Dairy
**Fruit**			
Canned fruit	125g	ADG	Fruit and vegetables
**Grains**			
Pasta and pasta-based products (incl. tinned spaghetti, instant pasta 'side dishes')	100g (1/2 cup cooked)	ADG	Grains (plain pasta) Mixed Dishes (pasta-based products or 'side dishes')
Rice and rice-based products (incl. flavoured pouches, instant rice 'side dishes')	125g (1/2 cup)	ADG	Grains (plain or seasoned pouches) Mixed Dishes (instant rice 'side dishes')
**Meat and meat alternatives**			
Beans, all types (incl. baked beans, refried beans)	150g	ADG	Protein
Fish, all types (incl. flavoured varieties)	100g	ADG	Protein
Meat (beef, lamb, pork, kangaroo), marinated meat and meat products (e.g., beef pie, puff pastry parcel)	75g	ADG	Protein Mixed Dishes (meat products)
Meat alternatives (all plant-based products)	150g	ADG (Meat and alternatives SS) Unified daily target lacking(1)	Protein
Nuts, all types (incl. flavoured varieties)	30g	ADG	Protein (plain nuts) Desserts (flavoured, coated nuts)

Poultry (chicken, turkey), marinated poultry and poultry products (e.g., chicken kiev)	80g	ADG	Protein
Processed meat (incl. ham, cabanossi, chorizo, salami)	50g (~2 slices)	ADG	Protein
Sausages	75g (~1 sausage)	ADG and review of product category SS	Protein
**Mixed foods**			
Baby food pouches	120g	Review of product category SS	Based on characterising ingredient/s
Dip	20g	Review of product category SS	Mixed Dishes
Pizza	125g	Review of product category SS	Mixed Dishes
Ready meals (frozen, chilled and shelf-stable)	350g	Wooldridge (2021)[2]<br>Gibson (2019)[3]	Mixed Dishes
Soup (canned)	350g	Considered a type of ready meal	Mixed Dishes
Soup (instant)	250ml<br>(soup reconstituted with water)	Standard SS across product category	Mixed Dishes
**Vegetables**			
Potato, all types and potato-based products (incl. chips gems, hash browns, scalloped potatoes)	75g (plain or lightly seasoned)<br>60g (potato-based products)	ADG	Fruits and Vegetables<br>Processed and packaged snacks<br>(all potato-based products)

* This is not an exhaustive list of all the SS used for HENRG categorisation in this study but includes food categories where the manufacturer SSs are wide ranging and variable.

† HENRG provide guidelines on classifications based on food type, but for more ambiguous foods, selection of the food category for classification was determined by the type and nature of the product. If the product was a whole food that was either flavoured (e.g., rice pouch) or marinated (e.g., meat) it was classified according to its whole food. Products that contained a combination of ingredients in significant proportions (e.g., meat pie, ready meals), were classified under ‘mixed dishes’.

(1). Marinangeli, C.P.F.; Curran, J.; Barr, S.I.; Slavin, J.; Puri, S.; Swaminathan, S.; Tapsell, L.; Patterson, C.A. Enhancing nutrition with pulses: Defining a recommended serving size for adults. Nutr. Rev. 2017, 75, 990–1006.
(2). Wooldridge, K.; Riley, M.D.; Hendrie, G.A. Growth of Ready Meals in Australian Supermarkets: Nutrient Composition, Price and Serving Size. Foods 2021, 10, 1667. https://doi.org/10.3390/foods10071667.
(3). Gibson, A.A.; Partridge, S.R. Nutritional Qualities of Commercial Meal Kit Subscription Services in Australia. Nutrients 2019, 11, 2679. https://doi.org/10.3390/nu11112679.
